# Predicting clinically unrecognized coronary artery disease: use of two- dimensional echocardiography

**DOI:** 10.1186/1476-7120-7-10

**Published:** 2009-03-06

**Authors:** Su Min Chang, Abdul Hakeem, Sherif F Nagueh

**Affiliations:** 1 Department of Cardiology, DeBakey Heart and Vascular Center, The Methodist Hospital, Houston, TX, USA; 2 Division of Cardiovascular diseases, University of Cincinnati, Cincinnati, OH, USA

## Abstract

**Background:**

2-D Echo is often performed in patients without history of coronary artery disease (CAD). We sought to determine echo features predictive of CAD.

**Methods:**

2-D Echo of 328 patients without known CAD performed within one year prior to stress myocardial SPECT and angiography were reviewed. Echo features examined were left ventricular and atrial enlargement, LV hypertrophy, wall motion abnormality (WMA), LV ejection fraction (EF) < 50%, mitral annular calcification (MAC) and aortic sclerosis/stenosis (AS). High risk myocardial perfusion abnormality (MPA) was defined as >15% LV perfusion defect or multivessel distribution. Severe coronary artery stenosis (CAS) was defined as left main, 3 VD or 2VD involving proximal LAD.

**Results:**

The mean age was 62 ± 13 years, 59% men, 29% diabetic (DM) and 148 (45%) had > 2 risk factors. Pharmacologic stress was performed in 109 patients (33%). MPA was present in 200 pts (60%) of which, 137 were high risk. CAS was present in 166 pts (51%), 75 were severe. Of 87 patients with WMA, 83% had MPA and 78% had CAS. Multivariate analysis identified age >65, male, inability to exercise, DM, WMA, MAC and AS as independent predictors of MPA and CAS. Independent predictors of high risk MPA and severe CAS were age, DM, inability to exercise and WMA.

2-D echo findings offered incremental value over clinical information in predicting CAD by angiography. (Chi square: 360 vs. 320 p = 0.02).

**Conclusion:**

2-D Echo was valuable in predicting presence of physiological and anatomical CAD in addition to clinical information.

## Background

Two-Dimensional echocardiography (2-D Echo) is well accepted for evaluation of cardiac function. [1] It is the most employed cardiovascular imaging modality for assessment of cardiovascular disease and is often performed in patients without history of coronary artery disease (CAD). It is well established that several echocardiograpahic measurements provide powerful prognostic information for cardiovascular outcomes such as presence of left ventricular hypertrophy, aortic sclerosis and LVEF. [1,2] However, the association of these features with underlying CAD is less well established. [3-5]

Although association of CAD and some isolated echo findings have been examined, no study have performed a direct comparison of different echo feature in predicting CAD in patients without history of CAD. Therefore, we sought to compare different echo findings to determine independent 2-D echo predictors of underlying anatomical CAD by angiographic coronary artery stenosis (CAS) and also physiological CAD by myocardial perfusion abnormality (MPA) by SPECT. Stress myocardial SEPCT imaging is the most commonly used imaging technique in assessment of suspected CAD. It provides high diagnostic accuracy for detection of angiographic CAD and adds incremental prognostic value over angiographic information. [6-8]

## Methods

### Study Population

We conducted a search of the cardiac imaging database of a large tertiary hospital to identify retrospectively 328 patients who had no known CAD, prior myocardial infarction or revascularization and underwent two -dimensional echo (TTE), stress myocardial perfusion with SPECT and coronary angiography. The tests (echo and SPECT) were ordered as per discretion of the treating physicians. The most common indications for SPECT were assessment of chest pain or CAD or preoperative evaluation.

The echocardiograms were performed within one year prior to stress myocardial SPECT. The indication for TTE was for assessment of left ventricular or valvular function. All patients underwent angiography within 3 months of SPECT. Patients were referred for coronary angiography by their treating physician based on the clinical presentation or SPECT findings.

Clinical characteristics and 12 leads ECG were prospectively collected at the time of SPECT. The clinical risk factors for CAD assessed were diabetes, hypertension, and hyperlipidemia, family history of CAD and history of smoking.

Abnormal ECG were defined as presence of any pathological Q waves, ST and T waves abnormalities, left ventricular hypertrophy, rhythm other than normal sinus rhythm and presence of AV nodal or bundle branch conduction abnormality.

### TTE Data

The TTE studies were performed with commercially available system (Acuson Sequoia C 256 or HP SONOS 5500). The studies were interpretated prior to SPECT by three expert level 3 echocardiographers who are board certified by the National Board of Echocardiography. Patient with prosthetic valves or severe valvular disease were excluded from this study. Two dimensional echocardiographic assessments were made using standard ASE recommendation. [9] The definition of echo abnormality is as followed:

-left ventricular enlargement (LVE): maximal LV end diastolic diameter > 50 mm at parasternal long axis view

-LV hypertrophy (LVH): LV septum and posterior wall > 1.2 cm by 2D measurement in parasternal long axis view in the absence of small left ventricular size

-presence of any wall motion abnormality (WMA) assessment as per ASE recommendation [9]

-LV ejection fraction (LVEF) > = 50% or < 50% by qualitative analysis or multiple diameter method [10]

-left atrial enlargement (LAE): 2 dimensional maximal volume of > 50 ml for male and > 45 ml for female by multidisks method at apical four chambers view [11]

-mitral annular calcification (MAC) as focal or diffuse echogenic structure located at the junction of the atrioventricular groove and posterior mitral valve leaflet on parasternal long, short axis or apical four chambers view

-aortic sclerosis (AS): focal area of increased echogenicity, thickening, or calcification of the aortic valve leaflets with peak aortic valve CW Doppler velocity < 2 m/sec for aortic sclerosis and > = 2 m/sec for aortic stenosis

### Gated Stress Myocardial Perfusion Imaging

Symptoms limited treadmill exercise using the Bruce protocol or four minutes adenosine stress imaging were performed as previously described. [12,13]All SPECT images interpretation and quantification was performed by two expert level 3 nuclear cardiologists. TTE results were not available for SPECT interpretation. The rest and stress polar maps were independently computer-generated and normalized through use of a circumferential profile analysis. The presence and extent of defect were quantified as previously described. [14] High risk MPA by SPECT was defined as >15% LV perfusion defect, LAD or multivessel distribution.

### Coronary Angiography

Qualitative visual analysis was used for coronary angiography evaluation. Coronary artery stenosis (CAS) was defined as presence of > 50% luminal diameter stenosis in one of the three major epicardial vessel or its major branches.

Severe CAS was defined as left main stenosis > 50%, 3 VD or 2VD involving proximal left anterior descending coronary artery.

### Statistical Analysis

Data are presented as mean ± SD. Chi-square or Fisher exact test was used for categorical variables comparison. All echo findings were presented as categorical variables. The two-sample unpaired Student's t-test was used to compare the continuous variables. Univariate analysis was used by comparing the presence of CAD with echo findings. Multivariable analysis using logistic regression was applied for the prediction of coronary artery disease. All collected variables were entered in the model at the same time. A p-value <0.05 was considered significant. Sigma Stat 3.1 was used for all analyses.

## Results

### Baseline Characteristics

Baseline clinical and echocardiographic characteristics are shown in Table 1.

**Table 1:** Baseline Clinical and 2-D Echo Characteristics

Mean Age (years)	62 ± 14
Age > 65 years	156 (48%)
Male gender	193 (59%)
Chest Pain as indication for SPECT	180 (55%)
DM	95 (29%)
Hypertesion	178 (55%)
Hyperlipidemia	77 (24%)
History of smoking	98 (30%)
Family history of CAD	98 (30%)
Number of Risk Factors	1.7 ± 1
Abnormal Rest ECG	245 (74%)
Unable to Exercise	109 (33%)
**2-D Echo Features**	
LVE	71 (22%)
LVH	170 (52%)
LVEF < 50%	69 (21%)
WMA	87 (27%)
LAE	174 (53%)
MAC	142 (43%)
AS	155 (47%)

DM: Diabetes Mellitus

LVE: left ventricular enlargement

LAE: left atrial enlargement

LVH: LV hypertrophy

WMA: wall motion abnormality

LVEF: LV ejection fraction

MAC: mitral annular calcification

AS: aortic sclerosis/stenosis

The group's mean age was 62 ± 14 years, 59% were men and 29% had history of diabetes mellitus. Forty-five percent of patients had 2 or more clinical risk factors.

Abnormal baseline ECG was present in 245 patients (74%) and in 55% of patients angina was the indication for SPECT. Pharmacologic stress in conjunction with SPECT imaging was performed in 109 pts (33%) due to inability to exercise. In the remaining subjects, treadmill stress testing was utilized with SPECT.

Myocardial SPECT and angiographic data are shown in Table 2. MPA was present in 201 patients (60%) of which, 137 were high risk. CAS by angiography was present in 165 patients (51%). Severe disease was present in 75 patients.

**Table 2:** Myocardial SPECT and Angiographic Data

SPECT		
Normal MPI	127	39%

Abnormal MPI	201	61%

High Risk Scan	137	42%

Predominant ischemic Defect	156	48%

Predominant Fixed Defect	45	13%

Perfusion Defect Size (% Left Ventricle)	19 ± 12	

**ANGIOGRAPHY**		

No significant CAS	163	49%

One vessel Disease	55	17%

Two vessels Disease without involving proximal LAD	35	11%

Two vessels Disease involving proximal LAD	19	6%

Three vessels or left main Disease	56	17%

MPI: myocardial perfusion imaging, CAS: coronary artery stenosis, LAD: left anterior descending artery

### 2-D echo predictors of CAD (Table 3)

**Table 3:** Correlation of 2 D Echo Features with MPI and CAS

		**Abnormal MPI**		P =	**CAS**		P =
**LVE**	**yes**	53/71	75%	**0.013**	39/71	55%	0.49
	**no**	148/257	57%		126/257	49%	
**LVH**	**yes**	90/170	53%	**0.002**	79/170	47%	0.18
	**no**	111/158	70%		86/158	54%	
**LVEF**	**<50%**	58/69	84%	**<0.001**	45/69	65%	**0.01**
	**> = 50%**	143/259	55%		120/259	46%	
**WMA**	**yes**	73/87	83%	**<0.001**	56/87	65%	**0.004**
	**no**	138/241	53%		109/241	45%	
**LAE**	**yes**	101/174	58%	0.24	74/174	42%	0.17
	**no**	100/154	64%		78/154	50%	
**MAC**	**yes**	110/142	77%	**<0.001**	87/142	62%	**<0.001**
	**no**	108/186	58%		77/186	42%	
**AS**	**yes**	105/155	68%	**0.003**	99/155	64%	**<0.001**
	**no**	96/173	55%		66/173	38%	

LVE: left ventricular enlargement

LAE: left atrial enlargement

LVH: LV hypertrophy

WMA: wall motion abnormality

LVEF: LV ejection fraction

MAC: mitral annular calcification

AS: aortic sclerosis/stenosis

2 D echo finding associated with presence of MPA and CAS based on univariables analysis is shown in table 2. LVE, LVH, EF < 50%, WMA, MAC and AS were predictors of perfusion defects by SPECT. On the other hand, LVEF < 50%, WMA, MAC and AS were predictors of CAS by angiography. Of those 87 patients who had WMA on resting echocardiography, 83% had MPA by SPECT and 78% had CAS by angiography. Sixty-nine patients had an EF < 50%. Of these, 84% had MPA by SPECT and 65% had CAS by angiography.

### Independent predictors of CAD

Multiple logistic regression analysis were performed for the entire cohort of patients. Age >65 yrs, male gender, inability to exercise, diabetes, WMA, MAC and AS were independent predictors of perfusion defects by SPECT and CAS by angiography (Table 4). Independent predictors of high risk MPA by SPECT and severe CAS by angiography were age, diabetes, inability to exercise and presence of WMA. (Table 5)

**Table 4:** Multivariate Logistic regression Analysis: Independent predictors of MPA by SPECT and CAS by angiography

		Odds Ratio	95% Confidence Intervals	P- value
**CAS**	age> 65	2.9	1.7–4.9	0.002
	Male	2.2	1.3–3.7	0.02
	Inability to exercise	2.9	1.5–5.5	<0.001
	DM	4.1	2.2–7.9	<0.001
	WMA	3	1.3–7.1	0.007
	MAC	2.2	1.2–3.7	0.03
	AS	2.3	1.3–3.9	0.03
**MPA**	age> 65	1.4	1–2.5	0.05
	Male	2.7	1.7–4.8	<0.001
	Inability to exercise	3.1	1.8–5.3	<0.001
	DM	2.1	1.1–4	0.02
	WMA	2.2	1.1–6	0.04
	MAC	2	1.1–3.5	0.03

DM: Diabetes Mellitus

WMA: wall motion abnormality

MAC: mitral annular calcification

AS: aortic sclerosis/stenosis

**Table 5:** Multivariate Logistic regression Analysis: Independent predictors of High risk MPA by SPECT and severe CAS by angiography

		Odds Ratio	95% Confidence Intervals	P
**Severe CAS**	age> 65	3.76	1.97–7.17	<0.001
	Inability to exercise	2.9	1.01–5.5	0.04
	DM	4.5	2.2–5.7	0.001
	WMA	2.4	1.02–5.7	0.04
**High Risk MPA**	age> 65	2.2	1.3–3.7	0.003
	Inability to exercise	1.7	1.0–2.8	0.05
	DM	2.8	1.5–5.0	0.001
	WMA	2.26	1.05–4.9	0.02
	MAC	1.9	1.08–3.3	0.01

DM: Diabetes Mellitus

WMA: wall motion abnormality

MAC: mitral annular calcification

All but one of 15 diabetic patients with age > 65 yrs, unable to exercise, with EF <50% and WMA had CAD by angiography.

### Incremental value of 2D echocardiographic findings (Figure 1)

**Figure 1:**
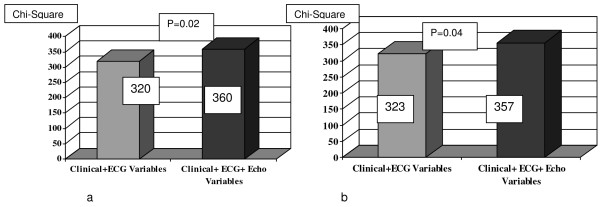
**Incremental value of 2D Echo Data over Clinical Information in Predicting CAD**. a: Coronary artery stenosis by CATH. b: Myocardial perfusion abnormality by SPECT.

2D echocardiographic findings had an incremental value over clinical information in predicting CAD by SPECT and angiography. (Chi square: 360 vs. 320 p = 0.02).

## Discussion

More than half of patients with major Q/QS wave's changes from the Cardiovascular Health Study did not report a previous myocardial infarction. [15] Echocardiography is often performed and abnormality found in patients without known CAD. Therefore it might offer a unique opportunity to identify patients with unsuspected CAD who might have a worse outcome and warrant early medical or invasive intervention. [2]

Previous studies have shown that some isolated echo features are associated with underlying CAD either using marker of CAD like SPECT, stress Echo or angiography. [16-18] However, very few of them performed a direct comparison of different echo features in predicting CAD in patients without history of CAD. To the best of our knowledge, current study is the first to correlate multiple 2 D echo findings to both physiological (myocardial perfusion SPECT imaging) and anatomical CAD (angiography). We showed that 2D echo information has indeed significant discriminative power in identifying underlying newly diagnosed CAD in addition to known clinical risk factors.

### Calcium deposit

Compared to the newer technology for detection of calcium such as cardiac CT, echocardiography is cheaper and there are no concerns for risk associated with ionizing radiation. Cardiovascular calcium deposits such as mitral annulus calcification and aortic sclerosis are thought to be a marker of generalized degenerative vascular process. They have been associated with a higher prevalence and incidence of coronary risk factors as well as CAD [19-21]. In one study, multiple calcium deposit (mitral annulus, aortic valve and aorta) have been associated with presence of abnormal myocardial perfusion imaging in patients younger than 65 years. However, patients with known CAD were not excluded, other echo findings were not examined and no angiographic data were reported. [18] In our study both mitral annulus calcification and aortic sclerosis were indeed associated with higher prevalence of myocardial perfusion abnormality and angiographic CAD. However their presence was not associated independently with more severe CAD when other echo abnormality such as WMA was present.

### Wall motion abnormality

The presence of resting WMA might represent underlying myocardial damage due to infarction, hibernation or stunning secondary to atherosclerotic CAD or non-ischemic insult to myocardium. [22-24] Therefore, although a resting WMA is associated with a higher cardiovascular event rate [25,26]; controversy still exists regarding the value of WMA in predicting CAD. [24] One recent study reported that resting WMA was predictive of abnormal myocardial perfusion by SPECT and ischemic response on dobutamine stress echo in 116 patients without known CAD or MI. [17]

Our results show that the resting WMA is indeed predictive of abnormal myocardial perfusion and obstructive coronary disease. Approximately 80% of these patients with WMA had CAD by SPECT or angiography. Moreover it is the only independent 2-D Echo predictor of high risk CAD. Indeed, in our study elderly diabetic patients unable to exercise with low EF and WMA appeared to be of highest risk of severe CAD.

Several of our patients (n = 11) have only fixed defect on SPECT but have no WMA on echo. This could be either due to attenuation artifact on SPECT or silent MI during time interval between performance of echo and MPI or angiography. In addition, it has also been reported that up to 30% of regions with fixed perfusion defect could have evidence of viability on echocardiography which has normal resting wall motion or resting hypokinesis worsened during exercise. [27]

### Left ventricular ejection fraction

Left ventricular ejection fraction is a well known prognostic marker for cardiovascular event. [3] But most of the adverse event is related to. In current study, it is associated with myocardial perfusion abnormality and angiographic CAD. However the association was no longer significant after adjustment for other clinical and echocardiographic factors such as wall motion abnormality. Several prior studies appeared to indirectly support our finding. In multivariable models, resting wall motion score index appears to be a more powerful predictor of combined cardiovascular event than LVEF in both post MI patients and patients evaluated for coronary artery disease [28,29]

### Left ventricular hypertrophy

Eccentric or concentric hypertrophy has been found to confer increased risk of incident coronary heart disease in The Framingham Heart Study and The Cardiovascular Health Study using M- mode echocardiography. [5,30] In our study the LV enlargement and hypertrophy were associated with perfusion abnormality but not with angiographic CAD. The discrepancy could be explained by different echo technique used and population studied. Also it is well known that non ischemic cardiomyopathy or hypertrophic cardiomyopathy could cause myocardial perfusion changes due to abnormal myocardial flow reserve in the absence of epicardial artery obstruction. [31,32]

### Left atrial enlargement

Left atrial enlargement have been shown to be a predictor of CV event in the The Cardiovascular Health Study. [30] However, except for higher risk of CHF, the association with other CV events is no longer present after adjustment of clinical risk factors. Theoretically left atrial enlargement could occur with increased filling pressure as a result of silent LV dysfunction secondary to CAD. However, it was not a predictor of CAD in our study. The explanation could be that LAE is more a direct indicator of underlying myocardial diastolic abnormality [33] rather than underlying atherosclerotic disease.

### Relation between echocardiography, myocardial perfusion imaging and angiography

Although the predictors of echo findings for either MPA or CAS were very similar, the predictive power was different. MPA correlates well with invasive angiographic findings but both false positive and negatives do occur. False positive tests could be due to attenuation artifacts, reduction of coronary flow reserve as in diabetics, hypertensives or hypertrophy with micro vascular disease in the absence of significant epicardial stenosis or non ischemic cardiomyopathy. [34,35] False negatives may be due to underestimation of flow heterogeneity or balanced ischemia. [36] However, it is well known that not all coronary stenosis are hemodynamically significant and may not induce perfusion abnormalities.

Several echo features provide powerful prognostic information in terms of cardiovascular outcomes such as death, CHF, infraction and stroke. However it is unclear if such abnormalities on echo are mere markers of other underlying disease or have direct pathogenic implication. By using intermediate cardiac endpoints such as presence of CAD, our findings provide some insight into the potential pathophysiologic mechanism of the association between echo finding and cardiovascular outcomes. Our findings appear to suggest that the adverse prognosis of wall motion abnormality and calcium deposits are probably more directly related to CAD whereas the chambers enlargement are probably more directly related to underlying myocardial systolic or diastolic dysfunction.

### Limitations

Our study has several limitations.

The retrospective nature of our analysis carried associated referral or selection bias. Patient was referred for TTE for assessment of LV and valve function and could have been referred for MPI when abnormal TTE finding was available to ordering physicians.

There was also a time interval between echo, MPI or angiography. We cannot exclude silent cardiovascular event or rapid progression of CAD during the time interval between TTE and MPI. Such progression could affect negatively the predictive power of echo findings. Our definition of LVH was based on increased wall thickness rather than LV mass calculation. However, in the CHS, increased LV wall thickness confers similar prognostic information as LV mass. [29] Detailed ECG analysis was not performed which could affect the predictive power of the echo finding. We performed our analysis using only binary variables and did not examine the potential correlation of different degree of LVEF, wall motion score and calcification with the severity of CAD. On the other hand, this simplified approach would be easier to apply in the clinical setting. Finally, outcome data were not available to explore the link between presence of CAD and adverse cardiovascular outcome associated with certain TTE abnormality.

### Clinical implications

Despite the predictive value of echo, we do not advocate use of TTE as screening test for unsuspected CAD. But when 2-D Echo is performed in patients without known CAD, we believe that certain scenario should prompt the clinicians to consider presence of significant underlying CAD and further diagnostic testing (such stress testing) might be warranted. 1) presence of depressed EF and WMA 2) presence of multiple calcification, in certain patient population (such as elderly, diabetic patients and unable to exercise)

## Conclusion

Several features in resting 2-D Echo provide incremental value over clinical information in predicting presence of physiological and anatomical CAD in patients without known CAD.

## Competing interests

The authors declare that they have no competing interests.

## Authors' contributions

SC and SN contributed to creation of the conceptual design, literature search, analysis of the data, interpretation of results, and writing of the manuscript. AH revised and edited the final manuscript.
